# Vaccine Hesitancy in Israel: Exploring the Relationships with Vaccination History, Knowledge, and Attitudes towards Influenza Vaccines

**DOI:** 10.3390/ejihpe14010003

**Published:** 2023-12-22

**Authors:** Keren Dopelt, Sophie Yukther, Tatyana Shmukler, Anuar Abudin

**Affiliations:** 1 Department of Public Health, Ashkelon Academic College, Ashkelon 78211, Israel; 2 School of Public Health, Faculty of Health Sciences, Ben Gurion University of the Negev, Beer Sheva 84105, Israel

**Keywords:** vaccine hesitancy, vaccination history, influenza, knowledge and attitudes toward vaccines, college students, Israel

## Abstract

Influenza vaccination is a highly effective strategy for mitigating all the repercussions of influenza infections. Despite the potential severity of influenza and the accessibility of secure vaccinations, worldwide rates of influenza vaccination continue to be low, particularly among students. This study examines the correlative relationships between influenza vaccine history, knowledge, attitudes toward influenza vaccines, and vaccine hesitancy among college students. To that end, we used an online questionnaire to conduct a cross-sectional study encompassing 610 students. A significant majority of participants reported having experienced influenza (82%), with slightly more than half having received influenza vaccinations in the past (57%). With respect to the current research year, health sciences students exhibited a higher likelihood of either having been vaccinated or intending to receive the vaccine than did their counterparts. Among students who had been vaccinated previously, approximately one-fifth opted for vaccination in the present year (21%). Similarly, 22% of the students whose parents were vaccinated chose to get vaccinated this year. Notable disparities in knowledge about influenza vaccines were observed across various departments, with health sciences students demonstrating the highest levels of awareness. Moreover, a negative relationship was found between knowledge, attitudes, and vaccine hesitancy. These results suggest that targeted lectures by professionals emphasizing vaccine safety and university-hosted events addressing this subject in collaboration with the Ministry of Health, incorporating influenza vaccination stations, could be instrumental in bolstering the vaccination rate.

## 1. Introduction

Influenza poses a notable public health threat, accounting for around 3–5 million severe cases and causing between 290,000 and 650,000 respiratory-related deaths annually worldwide [1,2]. The influenza vaccine is one of the most effective tools for mitigating the health-related, social, and economic effects of influenza [3]. It can prevent disease and an overload of the healthcare system [4]. The World Health Organization (WHO) recommends receiving a yearly influenza vaccination because of ongoing genetic alterations in the influenza virus [5]. Despite the severity of influenza infections and the availability of safe vaccines, vaccination rates are low globally, contributing to the burden on healthcare systems worldwide [6]. Influenza vaccination coverage among students falls behind in comparison to other age groups and continues to stay significantly under the goal [7]. Vaccination rates reported vary between 9 and 30%, and yearly outbreaks of the influenza virus result in severe disease that can be fatal even among students [8,9].

The Israeli Ministry of Health recommends that the entire population over six months of age be vaccinated against influenza every year before the onset of winter at no cost. However, in the influenza season of 2019–2020, roughly 25% of the Israeli population was vaccinated [10]. Studies have shown that seasonal influenza vaccination rates among the student population are low, ranging from 12 to 30% [11]. A study conducted by the National Foundation for Infectious Diseases (NFID) among students in the United States revealed that although 70% of students believed in vaccinating against influenza, only about 46% reported getting vaccinated [12]. The combination of crowded living spaces and frequent social interactions on campuses facilitates the rapid spread of influenza. This illness significantly hampers students’ academic performance and attendance, leading to heightened reliance on health services and increased prescription drug usage [13]. Students interact with family members and the community through their work, presence in fitness centers, and social events, and can thus be a source of outbreaks within the community [14]. Investigations of outbreaks among subgroups of students revealed high infection rates of up to 73% [11]. While influenza-related hospitalization is relatively rare among students, the potential burden this virus imposes on the student population is significant [11].

Vaccine hesitancy is defined as a combination of beliefs, attitudes, and behaviors exhibited by the general population and healthcare professionals regarding vaccinations, resulting in reduced vaccine coverage and an increased risk of preventable disease outbreaks [15]. The WHO designated vaccine hesitancy as one of the ten major global health concerns [5]. It is believed that vaccine hesitancy is responsible for diminishing vaccine coverage and the growing risk of outbreaks of diseases that can be prevented through immunizations [16,17]. With the ongoing increase in vaccine hesitancy and refusal rates globally, the protection against vaccine-preventable diseases provided by immunizations continues to decline [18]. Vaccine hesitancy is a complex phenomenon influenced by scientific, economic, socio-cultural, psychological, and political factors [19]. The threat of vaccine hesitancy persists despite significant clinical evidence supporting the benefits and importance of vaccines in preventing the spread of diseases [20].

The reasons for delaying or refusing vaccinations are complex and highly variable. The rapid development of vaccines and the pharmaceutical industry’s commercial interests have heightened public concerns and may influence decision making [21]. Studies of healthcare system employees have revealed high levels of vaccine hesitancy, even among doctors [21]. Doctors from Belgium, Austria, and other countries have voiced their reservations publicly regarding the influenza vaccine. Similarly, surveys have shown high levels of vaccine hesitancy among academics. A recent survey conducted among medical students showed that 23% of the respondents were disinclined to receive a COVID-19 vaccine, even if it received FDA approval [22]. A survey conducted among university lecturers and students in Indonesia indicated that half of the participants showed their willingness to receive the COVID-19 vaccine, while 11% expressed reluctance, and 39% were uncertain about getting vaccinated [23].

The reasons for influenza vaccine hesitancy among students have not been sufficiently researched. Documented obstacles include vaccine inaccessibility, a perceived lack of necessity, low motivation to vaccinate, and a lack of knowledge about the vaccine [24]. Casting doubt on vaccine efficacy and beliefs that the vaccine may have dangerous side effects reinforces the perception that it is possible to contract influenza from the vaccine [25]. A large public university study in the United States detected higher vaccine acceptance rates among students with a history of childhood vaccinations. Additionally, when unvaccinated students learned how the influenza vaccine protects healthy young individuals, most expressed increased willingness to get vaccinated [26].

Although vaccine hesitancy has undergone extensive research within the general adult population, there has not been a concentrated effort to encourage vaccination and public health communication specifically tailored to young adults. Motivating students to receive the influenza vaccine is challenging [12,27]. Generally, students tend to view themselves as healthy and having a low susceptibility to illness, despite the rapid spread of the influenza virus in areas surrounding campuses. In fact, low seasonal influenza vaccination rates among students are a global phenomenon [28]. Students with influenza immunity also protect their peers, family members, high-risk population groups, and others in the community [29]. Therefore, increasing vaccination rates among students in higher education institutions will help enhance overall coverage and achieve herd immunity against seasonal influenza [30].

Students represent an interesting group for research focused on investigating vaccine hesitancy, as they are considered educated, broad-minded, and aware of the perceived threat to humans from infectious diseases, constituting a special category of the young population who are open-minded and capable of responding quickly to public health issues [31]. Therefore, understanding the factors contributing to vaccine hesitancy among students may enable the development of a tailored plan to increase influenza vaccination rates. As such, this study aimed to determine whether there are correlations between influenza vaccination history, knowledge, attitudes towards influenza vaccination, and influenza vaccine hesitancy among college students.

## 2. Materials and Methods

### 2.1. Research Procedure

A cross-sectional analysis was conducted among the approximately 4200 students from Ashkelon Academic College. Approval for the study was obtained from the Ashkelon Academic College Ethics Committee (approval #42-2023). The survey questionnaires were developed using Qualtrics (Qualtrics, Provo, UT, USA) and were distributed to the students via email on 2 April 2023. After three weeks, a reminder prompting the completion of the questionnaire was sent using the same method. On 12 May 2023, the questionnaire was closed to further participation, coinciding with the end of the Israeli vaccination season. On average, participants took 5 ± 1.44 min to answer the questionnaire. The questionnaire began with an explanation of the research objectives and purpose and promised anonymity. Submitting the completed questionnaire signified the students’ informed consent to participate in the survey. Students could halt their responses anytime, and no questions were mandated. Out of the research population, a total of 703 individuals participated in the questionnaire, among which 610 students completed at least 90% of the questionnaire, representing 15% of the research population. This completion rate resulted in an overall response rate of 87% among those who responded.

### 2.2. Tools

A professional translator conducted the translation of the anonymous, closed, and self-administered questionnaire from English to Hebrew. Subsequently, it was distributed among 10 students who were not affiliated with the Ashkelon Academic College to verify the comprehensibility of the questions. Based on their feedback, the questionnaire underwent revisions. Furthermore, one specialist in public health and epidemiology, along with one expert in infectious diseases, assessed the questionnaire’s validity using the content validity method. The questionnaire comprised the following sections:

1. Demographic information: sex, age, marital status, religion, department, and year of study.
2. Vaccination and vaccination history: this included questions derived from Ryan et al. [26]: “Have you ever had the flu?”, “Have you ever been vaccinated against the flu?”, “Do your parents usually get vaccinated against the flu?”, and “Have you been vaccinated against influenza this year?”
3. Vaccine hesitancy: six questions were extracted from Silva et al. [32]. Participants were prompted to express their level of agreement with each statement in the questionnaire using a Likert scale, graded from 1 (not at all) to 5 (strongly agree), with an option to respond with “I don’t know”. The average score for each participant was computed, wherein the scales for questions 1 and 6 were reversed, and responses of “I don’t know” were excluded. A higher score signifies increased vaccine hesitancy. Cronbach’s α for reliability was α = 0.77.
4. Attitudes regarding influenza vaccines: this comprised five questions adapted from Silva et al. [32], prompting respondents to indicate their agreement with the provided statements using a Likert scale spanning from 1 (not at all) to 5 (strongly agree), with an additional option of “I don’t know”. The average of responses was computed for each participant. A higher score signifies a more favorable attitude toward influenza vaccines. Cronbach’s α for reliability was α = 0.74.
5. Knowledge about influenza and influenza vaccines: this consisted of 10 questions adapted from Ryan et al. [26], where respondents were prompted to state whether they believed each statement was correct, incorrect, or if they were uncertain. While Ryan et al. addressed each question separately, we created an aggregate variable in this manner: the total count of correct responses for each statement was tallied to compute the knowledge score. The original questionnaire contained 11 questions, and we utilized 10 questions, as the question ‘The intranasal influenza “spray” vaccine (FluMist) contains live attenuated virus’ is not relevant to Israel. The questionnaire underwent content validation by an infectious disease specialist.

### 2.3. Data Analysis

The data were analyzed using SPSS 29.0 (IBM, Armonk, NY, USA). Pearson correlation analyses were utilized to explore the relationships among the variables. Differences between groups of students were analyzed using χ^2^ tests, *t*-tests for independent samples, and one-way analyses of variance (ANOVAs). A linear regression model was employed to assess the prediction of vaccination hesitancy levels. All presented *p*-values resulted from two-sided tests and were deemed significant if they fell below 0.05.

## 3. Results

### 3.1. Participant Characteristics

A total of 610 individuals took part in the study, with women comprising 60%, while 53% reported being in relationships and 21% indicated having children. The majority of participants identified themselves as Jewish (83%). Almost half of the respondents were enrolled in the Faculty of Social Sciences (46%), followed by 35% in health sciences, and 19% in computer science and management. The average age of the respondents was 27.64 ± 7.20 years. A summary of participant characteristics is presented in Table 1.

### 3.2. Influenza Vaccination

Participants were asked about their history of influenza vaccination, whether they intended to get vaccinated this year, and the vaccination status of their parents and children. As shown in Table 2, most participants had experienced influenza infections at some point (82%; 89% when excluding participants who could not remember). Over half had previous vaccination records (57%; 61% when excluding participants who could not remember), and a similar percentage reported at least one parent having been vaccinated this season to the best of their knowledge. Over one-third of respondents who were parents reported vaccinating their children (38%). Among study participants, 12% were vaccinated, 44% intended to get vaccinated, 8% were undecided, and 36% did not intend to get vaccinated.

No significant differences were observed among faculties concerning vaccination history, parental vaccination, or children’s vaccination rates. However, significant differences were detected between faculties in terms of vaccination in the research period year (χ^2^ = 24.66, *p* < 0.001). Health sciences students showed a higher percentage of past vaccination (16%) and a greater intention to receive vaccination (47%) compared to computer science and management students (14% and 52%, respectively) and social sciences students (11% and 35%, respectively).

### 3.3. Associations between Vaccination History, Parental Vaccination, and Present Vaccination Status

The associations between the history of influenza vaccination, parental vaccination, and influenza vaccination in the current year were examined using chi-square tests after excluding participants who responded “do not remember”. Significant differences were observed between students who had received vaccinations in the past and those who had not been vaccinated against influenza in the present year (χ^2^ = 55.81, *p* < 0.001). Among those students who received vaccinations in the past, 21% were vaccinated in the current year, while 30% indicated that they did not intend to get vaccinated, compared to students who had not been vaccinated against influenza in the past, among whom only 1% were vaccinated in the current year, with 38% declaring that they had no intention of getting the vaccine.

Significant differences were also detected between students whose parents were vaccinated and those with only one vaccinated parent with respect to the influenza vaccination rate for respondents during the current year (χ^2^ = 15.55, *p* = 0.001). Among students for whom both parents were vaccinated, 22% were vaccinated this year, compared to just 11% of students with only one vaccinated parent.

### 3.4. Levels of Knowledge, Attitudes, and Vaccine Hesitancy

Table 3 presents the levels of knowledge and attitudes toward influenza vaccines and vaccine hesitancy. Overall, the knowledge about and attitudes toward influenza vaccines among study respondents were relatively low, while the level of vaccine hesitancy was moderate.

### 3.5. Relationships among Knowledge, Attitudes, and Vaccine Hesitancy

Relationships among variables were evaluated through Pearson correlation analyses. We detected significant negative associations between the level of knowledge, attitudes, and vaccination hesitancy (r_p_ = −0.35, *p* > 0.001; r_p_ = −0.43, *p* > 0.001, respectively). This indicates that a higher level of knowledge and more positive attitudes towards influenza vaccines are associated with lower levels of vaccine hesitancy.

### 3.6. The Relationships between Vaccination History and the Study Variables

The differences between students who had and had not been vaccinated in the past in relation to the study variables were tested using independent sample *t*-tests. Significant differences were observed between these groups regarding their levels of knowledge (*t* = 6.50, *p* < 0.001), attitudes (*t* = 3.24, *p* < 0.001), and vaccination hesitancy (*t* = 6.69, *p* < 0.001). Specifically, students previously vaccinated demonstrated higher knowledge levels (4.62 vs. 3.32), more positive attitudes (2.96 vs. 2.69), and reduced vaccination hesitancy (2.95 vs. 3.23) relative to unvaccinated students.

### 3.7. Differences between Faculties

Differences between faculties were examined using one-way ANOVA. Significant differences were found between faculties in terms of knowledge (F_(551)_ = 7.55, *p* < 0.001). Students in the health sciences faculty demonstrated the highest knowledge level, followed by students in the social sciences and, finally, students in computer science and management (averages of 4.62, 3.87, and 3.67, respectively). Scheffe post hoc tests revealed that students in the health sciences faculty exhibited significantly higher knowledge levels than students in either of the other faculties.

Significant differences were also detected among faculties in terms of attitudes toward influenza vaccination (F_(566)_ = 16.37, *p* < 0.001). Students in the health sciences faculty showed the highest level of confidence, followed by students in computer science and management, and, finally, students in the social sciences (averages of 3.05, 2.95, and 2.56, respectively). Scheffe post hoc tests indicated that students in the social sciences faculty held significantly more negative attitudes than those in the health sciences and computer science and management faculties.

Lastly, significant differences emerged across faculties concerning influenza vaccine hesitancy levels (F_(565)_ = 3.17, *p* < 0.05). Notably, students in computer science and management reported the highest hesitancy level, succeeded by social sciences students, and, lastly, health sciences students (averaging 3.22, 3.10, and 3.00, respectively). Subsequent Scheffe post hoc examinations indicated significantly greater hesitancy levels among computer science and management students compared to those in the health sciences faculty.

### 3.8. Hierarchical Linear Regression Model for the Prediction of Influenza Vaccine Hesitancy

The results of hierarchical linear regression models developed to predict influenza vaccine hesitancy are presented in Table 4. In the final model, which includes all the variables found to be significant in the previous models, the predictive ability of sex, religion, year of study, vaccinated parents, and knowledge level was preserved. Knowledge level, year of study, and previous immunization were all found to strongly predict vaccine hesitancy. The explained variance of the final model was approximately 21% (*p* < 0.001).

## 4. Discussion

Half of the participants in the current study had been vaccinated against the influenza virus prior to the study period. For at least half of the participants, at least one parent had been vaccinated against the virus, and among the parents of the children, about one-third of their children had received a vaccine against the virus. Among study participants, about 10% had been vaccinated against the virus, while approximately 44% planned to get vaccinated; the remainder were undecided or did not plan to get vaccinated. Generally, students perceive themselves as healthy individuals with a lower chance of contracting infectious diseases; as a result, their vaccination rate is low. Similar findings have also been reported in studies conducted throughout the world aimed at clarifying the percentage of students who were vaccinated against influenza. In these studies, 10–30% of students were found to have been vaccinated against the virus [11,33].

In contrast to these prior results, a study conducted by the NFID [12] among students in the United States found that 46% reported having been vaccinated against influenza. Among those students who had not been vaccinated, about 77% expressed a positive intention to get vaccinated. It is possible that since the healthcare system in the United States is private and disease treatments are costly (cost of doctor visits, medications, potential hospitalization), the vaccination rate is higher compared to countries where public healthcare services are more prevalent to avoid paying for treatment.

With respect to vaccination during the year of the research period, significant differences were found between faculties in the present study, with more students from the health sciences having been vaccinated or planning to be vaccinated compared to computer science and management or social sciences students. Similar findings were obtained in previous studies showing that influenza vaccination was more common among medical students than students in other professions [26,34]. This may be because health sciences students often have clinical experience in hospitals and clinics, making them more concerned about the risk of infection. In some cases, these students are required to be vaccinated to begin their clinical work out of concern for themselves, patients, and the teams with which they interact.

When comparing students who were vaccinated in the past to those who were not, a significant difference in vaccination was also found with respect to vaccination status during the research period year. Among students who were previously vaccinated, more stated that they intended to get vaccinated this year than those who were not vaccinated. These findings align with studies that explored factors related to positive intentions to get vaccinated against influenza. In these studies, students who planned to get vaccinated in the future were mostly those who had already been vaccinated in the past [35]. Similar findings were found in a study by Ryan et al. [26], which saw a higher vaccination rate among students with a childhood vaccination history. Similar findings were also obtained in studies conducted in other research populations, showing that previously vaccinated participants were more likely to receive or plan to receive their future vaccinations [36,37].

Additionally, a significant difference was found in vaccination intentions depending on the number of vaccinated parents. Among students with two vaccinated parents, 22% had been vaccinated in the current year, as compared to just 11% of students with only one vaccinated parent. These findings align with the existing literature indicating that when children receive support from their parents regarding vaccines, or when their parents support vaccinations, in most cases, they too will receive the vaccine. This can explain the finding that the higher the vaccination rate among parents, the higher the likelihood that the child will be vaccinated [38]. Another prior study also supported the notion that the higher the vaccination rate among family members and friends, the higher the likelihood that the individuals would receive it [39].

The present findings highlight differences between students who had previous vaccination records and those who had never received vaccination concerning the study variables. Students who had previously been vaccinated showed higher knowledge levels, more positive attitudes, and lower hesitancy than students who had never been vaccinated. These differences can be explained using the theory of planned behavior, which suggests that a person’s behavior is influenced by their attitudes toward the vaccine. According to this theory, someone with a more positive attitude toward the vaccine is more likely to choose to receive it. Additionally, someone who has already received the vaccine is expected to have positive attitudes that initially motivated them to receive it [40]. Similar findings were obtained in another study conducted among students in the United States, where it was found that previously vaccinated students are more likely to get vaccinated and to have higher levels of knowledge and positive attitudes on the subject [26]. Duradoni et al. [41] explained that attitudes are influenced by various life experiences, and these attitudes influence behavior.

Negative correlations were observed between knowledge and both attitudes toward influenza vaccines and vaccine hesitancy. Similar findings have been reported in studies that examined factors related to vaccine hesitancy. For example, a lack of knowledge was identified as an influencing factor in vaccination intentions among students in the United States [24]. In a study by Ryan et al. [26], the relationship between knowledge, attitudes, and vaccine hesitancy was examined, revealing significant correlations. A survey conducted among second-degree nursing students in Italy further found that as knowledge levels and attitudes became more positive, vaccine hesitancy decreased. In this study, the researchers also expanded and included students in intervention programs to promote vaccination. They found that students involved in the program had increased knowledge levels and reduced vaccine hesitancy [42]. According to Nerini et al. [43], individuals who held the belief in the vaccine’s efficacy and had increased confidence in their ability to receive it were more inclined to engage in the vaccination process. Notably, fear was linked not just to the anticipation of adverse health effects but also to its impact on one’s social interactions.

Generally, the level of knowledge regarding influenza vaccinations in this research was found to be low (an average of 4.04 ± 2.39 out of 10). Many students did not know the answers to most knowledge questions. In the research conducted by Sandler et al. [44], a low level of knowledge was also found regarding influenza vaccines and their effectiveness. In addition, differences were found between health science students and other students in terms of the analyzed study variables. Health science students had the highest level of knowledge, with these levels being markedly higher than those of social science or computer science and management students. This finding is logical, given that health science students acquire relevant knowledge about vaccines as part of their comprehensive training. Therefore, there is a strong likelihood that their knowledge levels will be higher than those of other students who do not acquire this knowledge in their studies [34]. Social science students had more negative attitudes toward vaccines than did health science or computer science and management students. Similar findings were obtained in a study conducted among students in Italy, which found that attitudes and vaccination intentions varied among students from different academic disciplines. These attitudes and intentions were generally more positive among medical students [35]. This may also be because medical and nursing students have more knowledge about vaccination topics, in large part because they deal with these subjects as part of their curriculum. Knowledge is a significant factor in this context, as has also been found in other studies conducted among students, with students who received knowledge about vaccines expressing more positive attitudes and intentions to get vaccinated after receiving relevant information [26,45].

Finally, significant differences were found between faculties in terms of influenza vaccine hesitancy. Computer science and management students had the highest hesitancy rates, followed by social science students and health science students. Similar findings have been reported in other studies that included students from various fields. For example, a study conducted in Saudi Arabia found that health sciences students had more positive vaccination intentions, consistent with lower hesitancy rates compared to students from other study areas [34]. Based on the research literature, vaccine hesitancy is ultimately reflected in the vaccination rate. Therefore, based on the vaccination rate of students from different study fields, it is also possible to learn about their hesitancy levels, assuming that higher hesitancy levels correspond to lower vaccination rates. In a study of 604 students from Hokkaido University in Japan, researchers examined the reasons related to the vaccination rate among students and found that the vaccination rate among health science students was three times higher than among students from other fields of study [46].

Furthermore, a hierarchical regression model was constructed based on the findings of this study in an effort to predict vaccine hesitancy among students. Shon et al. [38] discovered a higher vaccination frequency among female students compared to male students, suggesting potentially elevated levels of vaccine hesitancy among male students, aligning with the current findings. Additionally, the results indicate that students with vaccinated parents displayed lower levels of vaccine hesitancy. This was also supported by the research literature, with several explanations for this finding having been advanced [26,38,39]. According to the comprehensive model, the level of knowledge was the most accurate predictor of vaccine hesitancy. Similar findings were obtained in other studies conducted on this subject involving students [24,26,47]. Vaccine willingness is essential among students and populations that are generally not at risk in order to increase vaccination rates and generate herd immunity that can help protect the entire population [48].

### Study Limitations

This study was conducted among students from only one college, which may affect the generalizability of the findings to all students in Israel. Additionally, most participants were not vaccinated against influenza in the study year, and over one-third did not intend to get vaccinated. These data may indicate a selection bias where vaccine-hesitant students were more likely to respond to the questionnaire. Moreover, due to the limitation of our scope and resources, we could not use antibody detection to cross-reference the participants’ responses regarding their history of influenza. In addition, it appears that there was a bias towards vaccine preference in the tool that assessed the ‘knowledge about influenza and influenza vaccines’ level.

## 5. Conclusions

These research findings emphasize the importance of studying the factors associated with vaccine hesitancy among students and increasing knowledge about influenza vaccine safety, especially among students who do not receive this information as part of their study program. Young individuals sometimes believe that their vaccination is not essential when, in reality, their failure to be vaccinated might hinder immunity coverage in their community. Based on the present findings, it is recommended that intervention programs be developed to increase vaccination rates among all students, especially those studying non-health-related professions. Rising vaccination rates can be achieved through targeted lectures on vaccine safety by professionals and organizing campaigns within the college, which could include influenza vaccination stations in collaboration with the Ministry of Health [49]. Additional interventions can include meetings between unvaccinated students and vaccinated parents. Messages encouraging vaccinations can be delivered through social media platforms used by young people, via network influencers, such as Instagram and TikTok.

Further research should be conducted to thoroughly clarify the factors related to vaccine hesitancy and how to overcome such hesitancy. Developing intervention programs that can help increase vaccine coverage is important, as is the evaluation of the most effective intervention strategies. Moreover, in future studies, a representative sample of students from various colleges and universities should be included to obtain a more comprehensive picture of vaccine hesitancy in this population. These studies should also be expanded to other populations such as the elderly, pregnant women, and parents of young children. It seems that expanding the scope of research on vaccine hesitancy to additional populations is important in order to understand the challenges in increasing flu vaccination rates among various demographics. Different types of interventions can be tailored to each population group. For elderly populations, emphasizing the risk of complications and the difficulty in recovering at an older age may be effective. Among pregnant women, highlighting the potential danger to the fetus could be crucial. For mothers of children, emphasizing the maternal concern and the fact that an ill child requires care and absence from work might be impactful. In essence, interventions aimed at promoting vaccinations need to be specific to the population type and stage in life.

## Figures and Tables

**Table 1:** Participant characteristics.

Characteristics	*N*	%
Sex		
Male	243	40
Female	367	60
In relationship	324	53
Have children	128	21
Jewish	509	83
Faculty		
Health Sciences	202	35
Social Sciences	262	46
Computers and Management	106	19
Year of studies		
1st	310	51
2nd	198	32
3rd and 4th	102	17

**Table 2:** Influenza vaccination responses (*n* = 610).

Question	Responses	*N*	%
Ever had influenza	Yes	501	82
	No	59	10
	Don’t remember	50	8
Vaccinated against influenza	Yes	351	57
	No	223	37
	Don’t remember	36	6
Parents vaccinated against influenza	Yes, both	197	32
	Yes, one of them	152	25
	Do not know	261	43
Participants vaccinated this year against influenza	Yes	76	12
	Intend to vaccinate	269	44
	Do not intend to vaccinate	217	36
	Undecided	48	8
Are their children vaccinated (*n* = 128)	Yes	32	25
	Some of them	17	13
	No	79	62

**Table 3:** Levels of knowledge, attitudes, and vaccine hesitancy.

Variables	Maximum Obtainable Score	Range Obtained by Respondents	Mean ± SD *
Knowledge about influenza vaccines	9	0–10	4.04 ± 2.39
Attitudes toward influenza vaccines	5	1.00–5.00	2.82 ± 0.97
Vaccination hesitancy	5	1.00–5.00	3.11 ± 0.70

* SD = Standard deviation.

**Table 4:** Hierarchical linear regression model results (adjusted values) for models designed to predict influenza vaccine hesitancy.

Variable	Demographic	Education	Vaccination	Research Variables	Combined Model
	β	β	Β	β	β
Sex _(0–male, 1–female)_	−0.13 **				−0.08 *
Age	−0.05				
Marital status _(0–yes, 1–no)_	−0.09 *				
Children _(0–yes, 1–no)_	0.07				
Religion _(0–Jewish, 1–not Jewish)_	0.11 *				0.09 **
Birth _(0–Israel, 1–abroad)_	0.05				
Year of study		−0.14 ***			−0.12 **
Health sciences _(0–no, 1–yes)_		−0.04			
Computer science and management _(0–no, 1–yes)_		0.06			
Had influenza _(0–no, –1–yes)_			0.05		
Parents vaccinated _(0–no, 1–yes)_			−0.17 ***		−0.08*
Knowledge				−0.26 ***	−0.31 ***
Attitudes				−0.02	
Adjusted R Square	0.03 ***	0.03 ***	0.06 ***	0.20 ***	0.21 ***
*n*	605	565	534	575	545

* *p* < 0.05, ** *p* < 0.01, *** *p* < 0.001.

## Data Availability

The data presented in this study are available on request from the corresponding author.
